# Supplementary material for: Quantitative mapping of protein-peptide affinity landscapes using spectrally encoded beads
Source: eLife. 2019 Jul 8;8:e40499. doi: 10.7554/eLife.40499 (PMC6728138; doi:10.7554/eLife.40499)
Supplement: Supplementary file 1. [file elife-40499-supp1.docx]

**Supplementary file 1**. **List of literature affinities and references.**

| Name | Sequence | K_d_ Range (µM) |
| --- | --- | --- |
| NFATc1 | ALE**SPRIEIT**SCLG | 2.5^1^-25^a,2^ |
| NFATc2 | SGL**SPRIEIT**PSHE | 6^3^ |
| AKAP79 | RME**PIAIIIT**DT | 0.4-1.5^4^ |
| RCAN | T**PSVVVH**VC | 1-45^3,5^ |
| PVIVIT | AGPH**PVIVIT**GPHEE | 0.5^4^ |
| A238L | FKKK**PKIIIT**GCE | 0.8^6^ |
| Notes: a. Value reported as IC_50_ | | |

**References**

1. Park, S., Uesugi, M. & Verdine, G. L. A second calcineurin binding site on the NFAT regulatory domain. *Proc. Natl. Acad. Sci.* **97,** 7130–7135 (2000).

2. Garcia-Cozar, F. J. *et al.* Two-site interaction of nuclear factor of activated T cells with activated calcineurin. *J. Biol. Chem.* **273,** 23877–23883 (1998).

3. Mulero, M. C. *et al.* Inhibiting the Calcineurin-NFAT (Nuclear Factor of Activated T Cells) Signaling Pathway with a Regulator of Calcineurin-derived Peptide without Affecting General Calcineurin Phosphatase Activity. *J. Biol. Chem.* **284,** 9394–9401 (2009).

4. Sieber, M. & Baumgrass, R. Novel inhibitors of the calcineurin/NFATc hub - alternatives to CsA and FK506? *Cell Commun. Signal.* **7,** 25 (2009).

5. Li, H., Rao, A. & Hogan, P. G. Interaction of calcineurin with substrates and targeting proteins. *Trends Cell Biol.* **21,** 91–103 (2011).

6. Grigoriu, S. *et al.* The Molecular Mechanism of Substrate Engagement and Immunosuppressant Inhibition of Calcineurin. *PLoS Biol.* **11,** e1001492 (2013).
