# Supplementary material for: Quantitative mapping of protein-peptide affinity landscapes using spectrally encoded beads
Source: eLife. 2019 Jul 8;8:e40499. doi: 10.7554/eLife.40499 (PMC6728138; doi:10.7554/eLife.40499)
Supplement: Supplementary file 2. [file elife-40499-supp2.docx]

**Supplementary file 2. Calculated savings and references vs other techniques.**

| Methods | Number of  peptides | Amount of  Protein (µg)^e^ | X Time  MRBLE |
| --- | --- | --- | --- |
| MRBLE | 1 | 32 | 1 |
| MRBLE | 96 | 32 | 1 |
| MRBLE | 384 | 32 | 1 |
| FP^a^ | 1 | 60 | 1.875 |
| FP^a^ | 96 | 5760 | 180 |
| FP^a^ | 384 | 23040 | 720 |
| SPR^b^ | 1 | 40 | 1.25 |
| SPR^b^ | 96 | 3840 | 120 |
| SPR^b^ | 384 | 15360 | 480 |
| GST^c^ | 1 | 20 | 0.625 |
| GST^c^ | 96 | 1920 | 60 |
| GST^c^ | 384 | 7680 | 240 |
| ITC^d^ | 1 | 800 | 25 |
| ITC^d^ | 96 | 76800 | 2400 |
| ITC^d^ | 384 | 307200 | 9600 |
| Notes: a. Fluorescence polarization^1,2^, b. surface plasmon resonance^3^, c. glutathione S-transferase pull-down^4,5^ d. isothermal titration calorimetry^6^, e. amount of protein estimated from published available protocols. | | | |

**References**

1. Li, H., Rao, A. & Hogan, P. G. Structural Delineation of the Calcineurin–NFAT Interaction and its Parallels to PP1 Targeting Interactions. *J. Mol. Biol.* **342,** 1659–1674 (2004).

2. Li, H., Zhang, L., Rao, A., Harrison, S. C. & Hogan, P. G. Structure of Calcineurin in Complex with PVIVIT Peptide: Portrait of a Low-affinity Signalling Interaction. *J. Mol. Biol.* **369,** 1296–1306 (2007).

3. Gill, H. S., Roush, E. D., Dutcher, L. & Patel, S. Direct Evidence for Calcineurin Binding to the Exon-7 Loop of the Sodium-Bicarbonate Cotransporter NBCn1. *Int. J. Biol. Sci.* **10,** 771–776 (2014).

4. Aramburu, Jose *et al.* Affinity-Driven Peptide Selection of an NFAT Inhibitor More Selective Than Cyclosporin A. *Science* **285,** 2129–2133 (1999).

5. Aramburu, J. *et al.* Selective inhibition of NFAT activation by a peptide spanning the calcineurin targeting site of NFAT. *Mol. Cell* **1,** 627–637 (1998).

6. Ma, Y. *et al.* Enzymatic and thermodynamic analysis of calcineurin inhibition by RCAN1. *Int. J. Biol. Macromol.* **72,** 254–260 (2015).
